# Book Reviews

**Published:** 1915-05

**Authors:** 


### BOOK REVIEWS

---

Books mentioned may be inspected at and ordered through this office. So far as possible, books received in any month will be reviewed in the issue of the second month following.

---

**Money in Goats**, W. Sheldon Bull, Buffalo, published by author. 50 cents, paper.

This is a considerably enlarged and thoroughly revised second edition. The history of goat raising is carried back to extreme antiquity. The book is copiously illustrated and takes up all practical features of this growing industry. It is significant that, while the importations of goat skins have declined from nearly 32 million dollars' worth in 1906, the exports of glazed kid have increased from less than two million to nearly seventeen million dollars' worth in 1914. Of a total of 579,617 carcasses inspected by the U. S. authorities, 1907-1914, with reference to their use as food, only 1350 were condemned, and of these 705 merely for emaciation. Not one case of tuberculosis was found. The goat is economic to raise, easily cleaned, cared for and milked, its size alone, rendering it available for use on small premises and by those unable physically to perform the manual work necessary to the care of cattle and horses. Thus, it not only offers a field for profitable employment to many persons unable to raise stock of large size and requiring large pasturage, but it is directly available wherever fresh milk is necessary as a matter of hygiene. Goat milk is pleasing to palate, and on account of the almost perfect immunity of this animal to tuberculosis, it should be available at low cost for infant feeding. Mr. Bull's efforts to stimulate the various phases of the goat industry deserve support from the medical profession. For some reason, perhaps our inclination to prefer bigness in this country, we have neglected this ancient industry. We strongly advise the medical profession, whenever beset with the problem of feeding children and invalids, and with special though not exclusive reference to tuberculosis, to endeavor to secure a local supply of goat's milk.

---

**Annual Report of the Battle Creek Sanitarium**, for the 48th year, 1913.

This institution has a staff of 26 physicians, surgeons and specialists; reports over 35,000 laboratory examinations, over 12,000 special examinations, over 255,000 special treatments and 1889 surgical operations. The number of patients treated has grown from 53 in 1866 to 5,693 in 1913, a total of nearly 90,000. Its work is based on such statistics as 138,000 urinary examinations, 37,000 of gastric contents, 79,000 of blood, 20,000 of faeces. It employed—and taught—85 nurses. Much other interesting information is contained in the report.

---

**Clinical Symptomatology.** Issued by the Purdue-Frederick Co., N. Y.

This is an interesting brochure, giving a table, alphabetically arranged, of principal groups of diagnostic points, for example: abdominal pain—at umbilicus casts—fatty, with refer ence to future tables. These tables apply to diseases of the heart, intestines, liver, lungs and pleura, nervous system, stomach, kidney, urine examinations, faeces, vomiting, sputum, exanthemata, each crosslined to show principal differential diagnostic features. The booklet is well worth perusal and filing for reference. If any physician has failed to receive one, he should apply to the company.

---

**Cystoscopy and Urethroscopy for General Practitioners.** Bransford Lewis, B. S., M. D., and Ernest G. Mark, A. B., M. D., with a chapter by Wm. F. Braasch, M. D. (St. Louis, Kansas City and Rochester, Minn., respectively). Published by P. Blakiston's Son & Co., Philadelphia. 113 illustrations, 23 in colors, 238 pages, $4.50.

The illustrations, X-ray reproductions, half tones and colored plates are very clear, possibly clearer and the tints brighter than will be encountered in actual practice by the general practitioner. However, the error if any is on the safe side, for it teaches what ought to be observed and fixes a mental picture that will compensate for slight errors of focussing, etc., by the comparatively inexpert. While the book deals mainly with technic, it is described both as to general principles and according to particular lesions and thus virtually includes a practical review of the clinical and pathologic features involved in the various diseases of the urinary tract. We rather question whether it is economically advisable for the general practitioner in the strict sense to attempt these refined methods of diagnosis and treatment or whether the best interests of patients would be conserved by encouraging a large number of men to acquire the necessary technical skill. But there is no reason why anyone of reasonable dexterity and likely to have a considerable experience along these lines should not take up this line of work, even if he does not expect to limit his practice. However this question be answered, the book itself is a treatise of the highest merit and even if one does not expect himself to enter into the practice of endoscopy of the urinary tract, it is worth while for the general practitioner to understand fully what can be expected in the cases which he may observe and which present problems of reference to the specialist.

---

**A Text-book of The Practice of Medicine.** For Students and Practitioners. By Hobart Amory Hare, B.Sc., M. D., Professor of Therapeutics, Materia Medica and Diagnosis in the Jefferson Medical College, Philadelphia; Physician to the Jefferson Medical College Hospital; one time Clinical Professor of Diseases of Children in the University of Pennsylvania.

Third edition, revised and enlarged. Imperial octavo, 969 pages, with 142 engravings and 16 plates in colors and monochrome. Cloth, $6.00, net. Lea & Febiger, Publishers, Philadelphia and New York, 1915.

This treatise follows the customary arrangements: general infections, diseases of particular systems of apparatus, and those rather vaguely classified as metabolic. The earlier editions have met with a favorable reception and the present edition has afforded opportunity for the revision always desirable in a progressive science and art, such as that of medicine. The work has been well done, all the better, we think, because no specious appeal has been made by departing from methods of arrangement and discussion which long experience has shown to be best.

---

**Medical Electricity and Rontgen Rays and Radium.**. By Sinclair Tousey, A. M., M. D., Consulting Surgeon to St. Bartholomew's Clinic, New York City. Second edition, thoroughly revised and enlarged. Octavo of 1219 pages, with 798 practical illustrations, 16 in colors. Philadelphia and London: W. B. Saunders Company, 1915. Cloth, $7.50 net; Half Morocco, $9.00 net.

This is a thorough treatise on the subjects included in the title. In the past, an exaggerated notion has prevailed as to the value of electricity especially Faradism, in therapeutics. Unfortunately, too, the first wave of enthusiasm as to electricity in medicine preceded the invention of instruments of adequate power, reliability and ultimate economy, this being especially the case with regard to the derivation of a Galvanic current from a series of wet cells. The natural tendency was to discard electricity as at best a placebo, or to lay undue stress on some particular form of current. The time has now come for a discussion of medical electricity, at once conservative and without going to the opposite extreme of therapeutic nihilism. Such a view point is assumed by the present author, with wide experience. A large part of the work is, however, devoted to the diagnostic use of X-rays, illustrated by beautiful plates which should stimulate the endeavors of those interested in this subject. At the same time, the therapeutics of the emanations, from the X-ray tube, from radium itself and its congeners, is well presented and in the same conservative spirit.

---

**Principles of Hygiene**: For Students, Physicians, and Health-Officers. By D. H. Bergey, M. D., First Assistant, Laboratory of Hygiene and Assistant Professor of Bacteriology, University of Pennsylvania. Fifth edition thoroughly revised. Octavo of 531 pages, illustrated. Philadelphia and London: W. B. Saunders Company, 1915. Cloth, $3.00 net.

Without attempting to prepare a work on sanitary administration or state medicine, or the chemic and bacteriologic basis of much that is included under the general term, hygiene is discussed broadly and facts of interest in the subject are not overlooked simply because their absence might be excused by the title. Air, ventilation, heating, water-supply, sewage, garbage, diet, exercise, clothing, personal, industrial, school, military and naval hygiene, soil, dwellings, methods of dissemination of disease—including the disposal of the dead—disinfection, quarantine, vital statistics are the principal chapter headings. The work should be used not merely by health officials and those technically interested in sanitation, but by the physician generally. It may even be suggested that indifference to a broad conception of his duties by the practitioner, manifested by a neglect of this subject more than any other, is leading to a curtailment of his field of usefulness and, therefore, to his influence in the community and even his prosperity.

